# New Antibiotics for Treating Infections Caused by Multidrug-Resistant Bacteria

**DOI:** 10.3390/antibiotics14100997

**Published:** 2025-10-05

**Authors:** Elisabete Machado, João Carlos Sousa

**Affiliations:** 1 UCIBIO-Applied Molecular Biosciences Unit, Laboratory of Microbiology, Department of Biological Sciences, REQUIMTE, Faculty of Pharmacy, University of Porto, 4050-313 Porto, Portugal; 2 RISE-Health, Faculty of Health Sciences, Fernando Pessoa University, 4200-150 Porto, Portugal; 3 Faculty of Health Sciences, Fernando Pessoa University, 4200-150 Porto, Portugal; jcsousa@ufp.edu.pt

**Keywords:** carbapenemases, β-lactamases, carbapenem-resistant *Enterobacterales*, carbapenem-resistant *Pseudomonas aeruginosa*, carbapenem-resistant *Acinetobacter baumannii*, MRSA, pre-XDR *Mycobacterium tuberculosis*

## Abstract

Infections caused by antibiotic resistant bacteria pose a serious threat to global health, leading to higher medical costs, longer hospital stays, and increased morbidity and mortality. An increasing number of bacteria have been implicated in untreatable infections due to multiple resistance mechanisms. In 2017, the World Health Organization published a list of the most important antibiotic resistant bacteria worldwide, for which there is an urgent need to develop new therapeutic options. In recent years, fortunately, new antibiotics have been approved for the treatment of infections caused by multidrug-resistant bacteria. The purpose of this review is to present the most impactful new antibiotics that are currently available for the treatment of these infections. The discovery of new therapeutic strategies will help to limit the spread of multidrug-resistant bacteria, but careful prescribing, appropriate use and monitoring of resistant strains will be crucial to ensure that they remain effective in the future.

## 1. Introduction

Bacteria resistant to three or more different classes of antibiotics (MDR, multidrug-resistant), to all but two or fewer (XDR, extensively drug-resistant), or even to all antibiotic categories (PDR, pandrug-resistant) [1] are constantly increasing, compromising the action of last-resort antibiotics (e.g., carbapenems, vancomycin). Therefore, it is not surprising the increasing number of infections and deaths in which they are implicated. Examples of these pathogens are *Klebsiella pneumoniae* resistant to third-generation cephalosporins and/or carbapenems, or MRSA (methicillin-resistant *Staphylococcus aureus*) [2]. They are typically resistant to multiple antibiotics from different classes. The rapid and worldwide spread of these bacteria poses a serious threat to global health, which becomes even more relevant in the scenario of increasingly slow progression in the discovery of new antibiotic molecules, and rapid emergence of bacteria resistant to each new antibiotic introduced [3]. These conditions create a perfect storm that could lead to a post-antibiotic era, in which a simple pharyngitis could once again be fatal, or in which surgery or organ transplants could no longer be performed with the success that is known today.

This scenario alerted different international health authorities for immediate action to combat this imminent threat. In view of this situation, the World Health Organization (WHO) created a list of multidrug-resistant bacteria that should be prioritized for investment in research and development of new antibiotics [4]. The list organizes these bacteria by different levels of priority, with those compounding the “Priority 1” group being of “Critical Priority”. To date, the three most important bacterial threats to human health for which the development of new antibiotics is critical, are the following Gram-negative bacteria: carbapenem-resistant *Acinetobacter baumannii*, carbapenem-resistant *Pseudomonas aeruginosa*, carbapenem-resistant and/or third-generation cephalosporin-resistant *Enterobacterales* [4]. Among the Gram-positive bacteria, *Staphylococcus aureus*, *Enterococcus* spp. and *Streptococcus pneumoniae* stand out as also responsible for global resistance challenges, integrating the “High Priotity” or “Medium Priority” groups for development of new antibiotics [4].

Since the publication of this WHO priority list in 2017, more than seventy new molecules have been reported as being in different phases of clinical trials [5], some of which having non-traditional mechanisms of action [5].

**Figure 1:**
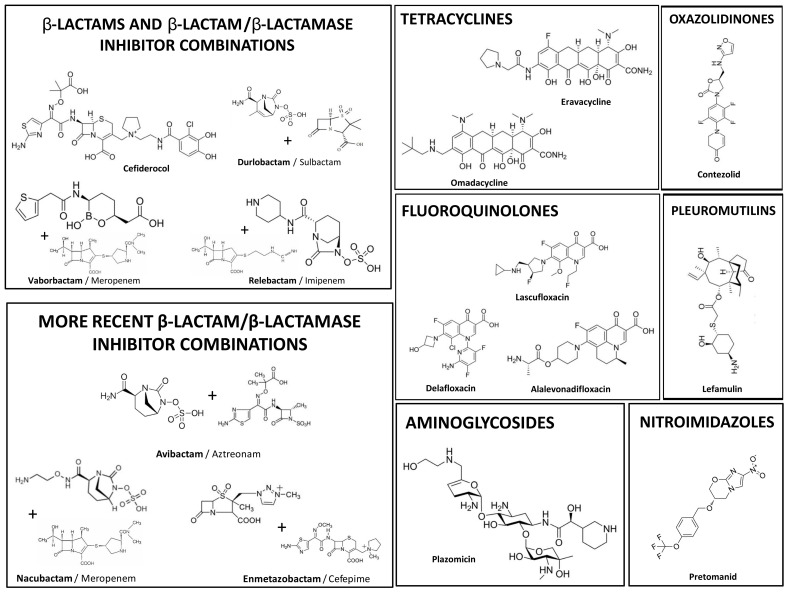
Chemical structures of new antibiotics or new antibiotic combinations approved by the Food and Drug Administration (FDA) for clinical use in infections caused by multidrug-resistant bacteria (new molecules are shown in bold).

As for new antibiotics or new antibiotic combinations with efficacy and safety already demonstrated by clinical trials and Food and Drug Administration (FDA)-approved for clinical use from 2017 to 2025, there are a total of sixteen molecules (Table 1, Figure 1) [5,6,7,8,9,10,11]. The expectation would be for new antibiotics with new mechanisms of action and/or new bacterial targets, but unfortunately this has not yet been accomplished [5]. Virtually all of them belong to previously approved classes of antimicrobials. Table 1 shows the name and class of these new antibiotics or antibiotic combinations, the priority multidrug-resistant bacteria for which they are approved, the mechanism of action, and the year of approval for clinical use [5]. Some of them are new molecules from the fluoroquinolones (delafloxacin, lascufloxacin, alalevonadifloxacin), or tetracyclines (eravacycline, omadacycline) classes. There are also representatives of β-lactamase inhibitors that by themselves do not have activity, but when used in combination with β-lactam antibiotics result in an effective therapy (e.g., vaborbactam with meropenem, relebactam with imipenem, avibactam with aztreonam, enmetazobactam with cefepime, and nacubactam with meropenem) [8,9,10,11,12]. A new cephalosporin (cefiderocol), a new aminoglycoside (plazomicin), a new pleuromutilin (lefamulin), and a new nitroimidazole (pretomanid) are also included. Additionally, there is a new oxazolidinone (contezolid) that was approved in China for the treatment of MRSA infections and received FDA-Qualified Infectious Disease Product (QIDP) and Fast Track designation [5,13]. The most recent approvals include an association of two β-lactamase inhibitors (sulbactam/durlobactam), and the antibiotic combinations aztreonam/avibactam and cefepime/enmetazobactam [6,7,8,9,14].

Since it is not possible to briefly analyze each of these new antibiotics, in this manuscript we will discuss in more depth examples of the most impactful new molecules approved for treating infections caused by MDR bacteria with higher clinical relevance, namely: carbapenem-resistant-*Enterobacterales* (CR-E), -*Pseudomonas aeruginosa* (CR-PA), and -*Acinetobacter baumannii* (CR-AB); MRSA; and pre-extensively-drug-resistant (pre-XDR) *Mycobacterium tuberculosis*. We aim to describe their main characteristics and provide information on their clinical use, in order to contribute to their judicious and appropriate use.

## 2. New Antibiotics Simultaneously Covering CR-E, CR-PA and CR-AB

CR-E, CR-PA, and CR-AB constitute the three microorganisms of maximum priority for the development of new drugs. Fortunately, a new antibiotic with action against all these three Gram-negative pathogens was recently approved for clinical use. It is a new β-lactam from the cephalosporins subclass, named cefiderocol. To better understand the importance of cefiderocol in the current epidemiological scenario of antibiotic resistance, it is important to highlight that β-lactams are antibiotics exhibiting high efficacy, low toxicity and a bactericidal effect by inhibiting the enzymes involved in the synthesis of the cell wall peptidoglycan [called PBPs (Penicillin-Binding Proteins)]. However, after years of exposure to this antibiotic class, bacteria developed diverse mechanisms of resistance to their action, the main of which amongst Gram-negative bacteria is the production of enzymes that inactivate β-lactams (β-lactamases) [15,16].

CR-E, CR-PA and CR-AB are resistant to carbapenems almost always due to the production of β-lactamases able to inactivate β-lactams of the carbapenems subclass, and hence designated carbapenemases. However, this problem is more serious, as carbapenemases can also inactivate other subclasses of β-lactams, such as penicillins, cephalosporins, and monobactams. Indeed, there is a high diversity of carbapenemases, which are distinguished according to their amino acid sequence and active site composition: class A, C, and D carbapenemases have serine in the active site, while class B carbapenemases (also called metallo-carbapenemases) contain a metal ion. Additionally, the different carbapenemase-types exhibit distinct profiles of β-lactam hydrolysis and inhibition by β-lactamase inhibitors. It is important to highlight that there are few β-lactam antibiotics and/or associations β-lactam/β-lactamasase inhibitors that still remain effective for treating infections by carbapenemase producers [17]. For metallo-carbapenemase producers, even combinations of β-lactams with β-lactamase inhibitors (such as the classic clavulanic acid, or even avibactam) will not be useful, being aztreonam the only β-lactam which could remain effective (in the past in association with ceftazidime/avibactam to overcome co-production of other carbapenemase-types, but in nowadays available in a combination with avibactam) [8,17]. Worsening this situation, CR-E, CR-PA and CR-AB are typically resistant to antibiotics from at least two other classes. These metallo-carbapenemase-producing pathogens represent, therefore, a particular clinical threat. In this context, the options are either to create antibiotics with new bacterial targets and/or new mechanisms of action or to design new molecules from already known classes of antibiotics, but with improved chemical structures that allow them to escape from several resistance mechanisms. The latter option was the possible one until date to combat those three critical priority pathogens. Cefiderocol is a new antibiotic from an already known antibiotic class and emerged for treating CR-E, CR-PA or CR-AB infections.

### Cefiderocol: A New Siderophore Cephalosporin

Cefiderocol is a new cephalosporin harboring multiple substituents that allow it to escape inactivation by β-lactamases, to have a good affinity for the target (PBPs) and to evade the various bacterial strategies to hinder its entry through the cell wall (Figure 1, Table 1) [18,19]. This highway for entry through the cell wall of Gram-negative bacteria results from the fact that the chemical structure of cefiderocol contains a catechol group that chelates iron ions (a siderophore group) [17]. This binding to iron allows cefiderocol to enter through iron transport systems, generally harbored by bacteria to capture this essential micronutrient. This circumvents problems of entry through the usual porin channels of the Gram-negative bacteria cell wall. Therefore, cefiderocol has the advantage of having an alternative mechanism for entry into the bacteria, exhibiting also higher affinity for the target (and hence higher antibacterial action) and stability against any of the bacterial β-lactamases known to date [19]. The entering of cefiderocol in such a camouflaged way through the iron transport systems present in the outer membrane of the cell wall of Gram-negative bacteria is a “Trojan Horse”-type approach [17]. This results in a spectrum of action on these three multidrug-resistant Gram-negatives (CR-E, CR-PA, CR-AB) that is not found in any other antibiotic, not even in combinations of β-lactams with new β-lactamase inhibitors [17,20]. Hence, the literature refers to cefiderocol as an “iron Trojan Horse”.

At present, the clinical use of cefiderocol is only approved for complicated urinary tract infections (UTIs) (including pyelonephritis) and healthcare-associated pneumonia [5,19]. It must be administered intravenously, and because it is eliminated by the kidneys, dosage adjustment is necessary in cases of renal failure. The main adverse reactions are at the gastrointestinal level (diarrhea, nausea and vomiting), as occurs with other β-lactams.

## 3. New Antibiotics for CR-E

The treatment of infections caused by CR-E can be implemented using other new therapeutic options besides the cefiderocol previously discussed, as eravacycline, meropenem/vaborbactam, imipenem/relebactam, aztreonam/avibactam, cefepime/enmetazobactam, meropenem/nacubactam or plazomicin (Table 1). Amongst them, one that deserves to be highlighted is plazomicin, due to its bactericidal effect and ability to bypass diverse aminoglycoside resistance mechanisms, and also because its mechanism of action extends the possibilities of coverage in case of infections involving CR-E producing different β-lactamase-types compromising most β-lactam therapies [17]. Despite the importance of plazomicin, the antibiotic combination aztreonam/avibactam will also be discussed, due to the revolutionary spectrum of this association over bacteria producing diverse carbapenemase-types.

### 3.1. Plazomicin: A New Aminoglycoside

Plazomicin is a new antibiotic from an already well-known class of antibiotics, the aminoglycosides. Aminoglycosides are polycationic antibiotics that are not orally absorbed and that act on bacterial ribosomes, more specifically on the 30S subunit. After binding, they cause a distortion of the ribosomes, which affects the codon-anticodon binding and leads to an incorrect reading of the genetic code. As a result, the bacteria produce abnormal proteins that after incorporation into the cytoplasmic membrane, create aqueous channels through which intracellular components responsible to maintain internal osmotic pressure can escape, resulting in bacterial death. Aminoglycosides are therefore bactericidal antibiotics. Bacteria have also developed different mechanisms of resistance to aminoglycosides, including alterations and/or protection of the target from the binding of the aminoglycoside, efflux pumps or permeability alterations preventing adequate intracellular concentrations, and enzymatic inactivation [16,21]. In this way, maintenance of the use of this antibiotic class will be increasingly based on resorting to new molecules, with well-refined chemical structures able to bypass diverse aminoglycoside resistance mechanisms.

Plazomicin is a new aminoglycoside (Figure 1, Table 1), which differs from all previous members of this antibiotic class due to its high stability against several aminoglycoside-inactivating enzymes (it is stable against 15 out of 17 of these enzymes) [19,22]. However, it can be expelled by non-specific efflux pumps present in *A. baumannii* and *P. aeruginosa*, and for this reason it is not used in infections caused by these pathogens. Since it is a polycationic antibiotic, it must be administered intravenously. Due to its polar nature, its distribution in tissues is also very irregular, with the exception of the renal cortex, where it reaches high concentrations. All these characteristics justify the indications of plazomicin for the treatment of complicated UTIs (including pyelonephritis) caused by CR-E [19]. Since it is eliminated via the kidneys, in the event of impaired renal function, it is imperative to adjust the dosage. It is also important to highlight that, similarly to classic aminoglycosides, plazomicin is nephrotoxic, ototoxic (might lead to irreversible hearing loss), may cause neuromuscular paralysis (rarely) and presents a risk to the fetus (mainly hearing loss).

### 3.2. Aztreonam/Avibactam: A Revolutionary β-Lactam/β-Lactamase Inhibitor Combination

This antibiotic combination is revolutionary in the way that it is active over CR-E producing diverse carbapenemases-types. Aztreonam is not inactivated by class B carbapenemases (metallo-carbapenemases), while it is hydrolyzed by class A and D carbapenemases. However, its association with avibactam, a β-lactamase inhibitor of class A and D β-lactamases, protects it additionally to these type of carbapenemases. The result is an antibiotic combination able to inhibit CR-E producing classes A, B and/or D carbapenemases [8]. It was approved by FDA for the treatment of complicated intra-abdominal infections (cIAI). In Europe it is additionally approved for the treatment of hospital-acquired pneumonia (including ventilator-associated pneumonia), complicated UTIs (including pyelonephritis), and for infections due to aerobic Gram-negative bacteria with limited treatment options [8].

Aztreonam/avibactam is of intravenous administration and requires dosage adjustment in case of renal impairment. Main adverse reactions include anemia, diarrhea, and increased hepatic enzymes (alanine aminotransferase and aspartate aminotransferase) [8].

The other antibiotic combinations, namely meropenem/vaborbactam, imipenem/relebactam and cefepime/enmetazobactam, have more limitations when comparing with aztreonam/avibactam. They are not able to remain stable against all carbapenemase classes, as occurs with aztreonam/avibactam, and hence their bacterial spectrum is not so extended (Table 1). In the case of cefepime/enmetazobactam its action is mainly against extended-spectrum β-lactamase (ESBL)-producing *Enterobacterales*, due to the higher inhibitory action of enmetazobactam over class A ESBL-type β-lactamases [9], despite recent clinical data support its potential role in inhibiting OXA-48 producers [9]. Meropenem/vaborbactam and imipenem/relebactam are mainly active against CR-E producing class A carbapenemases, due to the inhibitory characteristics of the β-lactamase inhibitors that compound these associations [17]. Finally, the meropenem/nacubactam antibiotic combination is still not FDA-approved but received FDA-QIDP and Fast Track designation. It includes nacubactam, a novel β-lactamase inhibitor with action against β-lactamases of classes A and C, and some of class D, being also able to inhibit the PBP2 involved in the synthesis of the bacterial cell wall peptidoglycan [10,11]. Hence, it has a dual mechanism of action that, when combined with meropenem, allows its action on CR-E producing class A or some class D carbapenemases [10,11].

## 4. New Antibiotics for MRSA

New antibiotics have also emerged for the treatment of infections caused by high-priority multidrug-resistant Gram-positive pathogens, such as MRSA. Examples are the fluoroquinolones delafloxacin, lascufloxacin and alalevonadifloxacin, and the tetracycline omadacycline [5,23]. Omadacycline has less severe adverse effects and exhibits very good oral bioavailability, allowing the transition from intravenous to oral administration with single daily dose regimens after hospital discharge, and for this reason it will be further discussed with more detail.

### Omadacycline: A New Generation Tetracycline

Similarly to other tetracyclines, omadacycline acts by binding to the bacterial ribosomes and preventing protein synthesis (Table 1). The binding occurs at the 30S ribosomal subunit (as for aminoglycosides), but specifically at site A, blocking the attachment of aminoacyl-tRNA to the mRNA-ribosome complex. This results in a reversible inhibition of the protein synthesis, and in a bacteriostatic action, in therapeutic doses [24,25]. Omadacycline was designed to escape from most of the tetracyclines resistance mechanisms developed by bacteria, as target changes and/or protection, and particularly efflux pumps, which constitute the main mechanism of resistance to tetracyclines [16,25,26,27,28]. In fact, omadacycline was especially designed to be unrecognizable by bacterial efflux pumps [28]. Only tetracycline inactivaction enzymes known as destructases would be able to restrain the action of this modern tetracycline [26,28].

In this context, omadacycline is part of the history of tetracyclines, a history that has been driven by the evolution of bacterial resistance mechanisms to these antibiotics over the last ten years [26,29]. However, the chemical structure of omadacycline (which is chemically an aminomethylcycline) (Figure 1) also allowed it to evolve into a tetracycline with both very good oral bioavailability and a half-life that allows it to be used under a single daily dose regimen. For all these reasons, omadacycline is classified as a new generation tetracycline [29].

Until now, the clinical use of omadacycline has been approved for infections caused by MRSA of this type: acute skin and skin structure infections, and community-acquired pneumonia [25,27,30]. Its good oral bioavailability and single daily dose facilitate the transition from intravenous to oral administration, and hence the continuity of the treatment after hospital discharge [25]. However, it is important to highlight that it is not a good option as monotherapy in immunocompromised patients, due to its bacteriostatic action. The patient should be advised to take omadacycline tablets with water and on an empty stomach after fasting for at least 4 h and not to consume products with multivalent cations (such as dairy products, antacids, iron-containing products), as they can form salts or chelates with omadacycline, reducing its absorption. Adverse effects are those frequently associated with tetracyclines, some of which are related to their affinity for calcification zones [29]. Tooth discoloration (with permanent brownish stains), hypoplasia of tooth enamel, and inhibition of bone growth (which is reversible) are the most common. In this way, omadacycline should not be used in pregnant women (especially in the second and third trimester of pregnancy), or in children up to 8 years of age.

## 5. New Antibiotics for Pre-XDR *Mycobacterium tuberculosis*

At the end of this brief review about some new antibiotics for priority multidrug-resistant bacteria, a new molecule will be addressed, which is already approved for the treatment of tuberculosis, the infectious disease that caused the most deaths before the COVID-19 pandemic [31]. This new molecule will be crucial for the treatment of tuberculosis caused by pre-XDR strains of *Mycobacterium tuberculosis* [32].

In order to better understand the importance of the new antibiotic that will be further discussed, it should be clarified that when the infectious agent of tuberculosis is resistant to rifampicin and isoniazid, it will be classified as MDR [32]. When it meets the definition of MDR and is, additionally, resistant to any fluoroquinolone, it will be classified as pre-XDR [32]. However, if a MDR strain is resistant to any fluoroquinolone and to another Group A drug (according to the WHO report [32], such as bedaquiline or linezolid), it should be classified as XDR and the chances of an effective treatment become substantially reduced [32].

The current situation is that whenever a patient has tuberculosis caused by a pre-XDR strain of *Mycobacterium tuberculosis*, or by a MDR strain but he does not tolerate or does not respond to conventional first-line antibiotics, one of the therapeutic regimens that is approved for clinical use is a 6-month triple therapy: bedaquiline, linezolid and pretomanid (a new antibiotic) [32]. This is a real treatment revolution compared to the duration of the classic treatment regimens for these cases.

### Pretomanid

Pretomanid is a new antibiotic for treating tuberculosis [33], which prefix “Preto-“ comes from “Pretoria”, the city in South Africa where it was developed [19]. Pretomanid belongs to the class of nitroimidazoles (like the well-known metronidazole) (Figure 1), and similarly to other antibiotics of this class, pretomanid requires bioactivation by reduction to become active (it is a prodrug). However, it has been shown to have an innovative dual mechanism of action in *Mycobacterium* spp. infections (Table 1) [19]. When the bacteria are at a high multiplication rate (as in the pulmonary alveoli, where there is high availability of oxygen), pretomanid acts by inhibiting the synthesis of mycolic acids (essential components of the cell wall of *Mycobacterium* spp.), resulting in bacterial death. Additionally, when *Mycobacterium* spp. is multiplying slowly in an anaerobic environment (as alveolar macrophages), the reactive nitrogen species (such as nitric oxide) generated by the activation of pretomanid, react with the cytochromes of the bacterial respiratory chain and reduce the levels of intracellular ATP, also compromising the bacterial survival. One disadvantage of pretomanid is that it is only useful for pulmonary tuberculosis, not being effective in cases of extra-pulmonary tuberculosis (representing only 5% of all forms of tuberculosis) [19]. Its half-life (t_1/2_) of 16-20h allows for a single daily dose regimen, which is comfortable for the patient [34]. As for adverse effects, it should be highlighted its hepatotoxicity, although this does not have major consequences when appropriately managed, being reversible [34].

## 6. Some Comments on Other New Antibiotics

Some new antibiotic molecules are not so impactful for treating infections caused by MDR bacteria with higher clinical relevance but deserve some comments due to their more favorable adverse effects profile, MDR bacterial spectrum and/or pharmacokinetic properties.

Contezolid, a new oxazolidinone that was approved for the treatment of MRSA infections, has a better safety profile and fewer adverse effects than linezolid, particularly regarding the myelosuppression and the monoamine oxidase (MAO) inhibition [13]. Moreover, dose adjustment of contezolid is not required in cases of mild renal or hepatic impairment [5,13].

The new antibiotic combinations sulbactam/durlobactam that was approved for the treatment of hospital-acquired bacterial pneumonia (HABP) and ventilator-associated bacterial pneumonia (VABP) caused by MDR *A. baumannii*, including CR-AB, constitute an advanced approach, as durlobactam is a diazabicyclooctane β-lactamase inhibitor with activity against Ambler classes A, C and D serine β-lactamases, protecting sulbactam from enzymatic hydrolysis and promoting its action against those MDR strains that are not important metallo-carbapenemase producers [6,7,14]. Dosage adjustment is necessary in cases of renal impairment.

Lefamullin, is the first systemically administered pleuromutilin approved for human use [5]. It is FDA-approved for the treatment of community-acquired pneumonia by *Streptococcus pneumoniae* non-susceptible to penicillin or *Haemophilus influenzae* ampicillin-resistant [5]. It could be administered orally or intravenously, and no dosage adjustment is required in patients with renal impairment.

## 7. New Antibiotics: Problems

Although the new antibiotics discussed here give some hope to the scientific community for treating infections caused by MDR bacteria, there are still some problems regarding their clinical use:

- Most have not been tested in clinical trials for use in less common severe infections (such as endocarditis, meningitis or osteomyelitis);
- There is a lack of data regarding their clinical use in special populations, such as children, the elderly, obese people and critically ill patients;
- Most of them belong to previously approved classes of antimicrobials, which increases the risk of rapid emergence and dissemination of bacterial strains harboring resistance mechanisms to those new recently approved antibiotics;
- They are expensive antibiotics.

Despite these shortcomings, there are situations in which these new antibiotics may represent the only and last alternative for treating infections caused by MDR bacteria. It is imperative to use them judiciously, so that they remain a long-term alternative.

Judicious use involves knowledge about the site of the infection, the etiological agent and the respective antibiogram (and, if possible, resistance mechanisms involved). This includes collecting clinical sample(s) from the patient and performing a laboratory diagnosis of the infection. All of this may delay the start of antibiotic therapy, but it will guide therapeutic decisions towards antibiotic therapy directed at the etiological agent of the infection and appropriate for the site of the infection.

The judicious use of these new antibiotics also requires updated information regarding epidemiological data on antibiotic resistance at local and regional levels, or even more. This is important especially in situations in which, after a clinical diagnosis of the infection, it is not possible to postpone the start of antibiotic therapy until the arrival of the microbiology laboratory results (e.g., meningitis, sepsis). Empirical treatment will have to be started. Therefore, if the epidemiological data corroborates that certain infections caused by MDR bacteria are uncommon in a given geographical area, it will not make sense to start empirical therapy with new antibiotics. On the contrary, if the local epidemiology is dramatic in terms of infections caused by MDR bacteria, it is justified to resort to these new alternatives in the therapeutic armamentarium.

It is also good clinical practice to revise all antibiotic treatments 48 to 72 h after starting, to check if the patient is improving clinically and/or if any changes need to be made to the initial therapy.

Finally, physicians should ensure that antibiotics are being used appropriately in terms of dosage, duration of treatment, and route of administration, among other aspects.

## 8. New Therapeutic Strategies

Bacterial resistance to antibiotics cannot be eliminated. It can only be slowed down. Therefore, besides the new antibiotics approved belonging to traditional antimicrobial classes and the few promising new pipeline antibiotics with novel mechanisms of action and/or targets (non-traditional antibiotics) [35,36], new strategies are needed to combat infections associated with MDR bacteria, namely non-antibiotic approaches (Table 2) [5,35,36]. Some new non-antibiotic approaches based on the use of antibodies, bacteriophages, phage-derived enzymes, and microbiome-modulating agents, amongst others, are already available [5,37,38].

Bezlotoxumab is an example of a new non-antibiotic strategy. This is an intravenous antibody that, when reaching the intestinal epithelium, binds to *Clostridioides difficile* toxin B and thus neutralizes its harmful effects on the intestinal mucosa [39,40]. As *Clostridioides difficile*-associated diarrhea is caused by toxins produced by the bacteria, instead by the bacteria itself, this approach using anti-toxin monoclonal antibodies could be useful. So far, in the clinical trials carried out, only bezlotoxumab has shown efficacy, so it is currently the only antibody approved for this clinical use, in combination with an antibiotic [39].

Another new strategy to combat infections caused by MDR bacteria appears to be the use of the so-called Direct Lytic Agents. These new compounds are proteins or peptides originally derived from the lytic systems of bacteriophages (viruses that infect bacteria), with unique mechanisms of action, based on a rapid destabilization of the cell wall that leads to bacterial lysis [41]. A compound of this type is already in Phase III clinical trials. It is exebacase, a direct lytic agent of the lysine class (hydrolytic enzymes of peptidoglycan, the main constituent of the bacterial cell wall) [42]. It has been tested in systemic infections caused by *S. aureus*, including endocarditis. Results from the first Phase III clinical trial were recently published and were unexpected, based on Phase II data establishing proof-of-concept for exebacase plus antibiotics in patients with MRSA systemic infections/endocarditis [43]. Future Phase III clinical trials with an enhanced design will elucidate its efficacy and determine whether it will be approved for clinical use.

## 9. Conclusions

Since 2017, sixteen new antibiotics or antibiotic combinations have been approved for the treatment of infections caused by MDR bacteria considered priority pathogens, in terms of the urgent need for new antibiotics. However, none of them have completely new mechanisms of action and/or bacterial targets. Moreover, although these new treatment strategies are emerging, there is still a lack of data on their efficacy and safety under specific conditions. The new promising pipeline antibiotics that are under research and development are also scarce, and only few non-antibiotic strategies are available.

Therefore, we must respectfully view this global threat to public health and use rationally the new traditional and future non-traditional antibiotic alternatives in the therapeutic armamentarium, while expecting for the demonstration of efficacy and safety in clinical practice of a sufficient diversity of non-antibiotic strategies. Meanwhile, it is imperative to implement targeted therapies and avoid empirical treatments. Only these actions could delay a near future entry into what many call the “post-antibiotic era”, which Alexander Fleming foresaw as early as 1946. Physicians and pharmacists, in the exercise of their profession, will certainly play an important role in addressing these issues, contributing to the protection of Public Health.

## Figures and Tables

**Table 1:** New antibiotics or new antibiotic combinations approved by the Food and Drug Administration (FDA) for clinical use in infections caused by multidrug-resistant bacteria.

Antibiotic/ Antibiotic Combination	Class	MDR Bacteria ^2^	Year of Approval	Mechanism of Action ^5^
Delafloxacin	Fluoroquinolone	MRSA	2017	Inhibition of bacterial DNA topoisomerase IV and DNA gyrase (topoisomerase II), disrupting DNA replication
Lascufloxacin	Fluoroquinolone	MRSA	2019	Inhibition of bacterial DNA topoisomerase IV and DNA gyrase (topoisomerase II), disrupting DNA replication
Alalevonadifloxacin	Fluoroquinolone	MRSA	2020	Inhibition of bacterial DNA topoisomerase IV and DNA gyrase (topoisomerase II), disrupting DNA replication
Meropenem/Vaborbactam ^1a^	β-Lactam (Carbapenem)/Boronate β-lactamase inhibitor	CR-E	2017	Inhibition of cell wall synthesis by blockage of PBPs (associated BLI protects from inactivation by class A β-lactamases)
Imipenem/Relebactam ^1a^	β-Lactam (Carbapenem)/Diazabicyclooctane β-lactamase inhibitor	CR-E	2019	Inhibition of cell wall synthesis by blockage of PBPs (associated BLI protects from inactivation by class A β-lactamases)
Aztreonam/Avibactam ^1a^	β-Lactam (Monobactam)/Diazabicyclooctane β-lactamase inhibitor	CR-E	2025	Inhibition of cell wall synthesis by blockage of PBPs without hydrolysis by class B β-lactamases (BLI protects from inactivation by class A and D β-lactamases)
Cefepime/Enmetazobactam ^1a^	β-Lactam (Cephalosporin)/Penicillanic acid sulfone β-lactamase inhibitor	ESBL-E	2024	Inhibition of cell wall synthesis by blockage of PBPs (associated BLI protects from inactivation by class A ESBL-type β-lactamases)
Meropenem/Nacubactam ^1a^	β-Lactam (Carbapenem)/Diazabicyclooctane β-lactamase inhibitor	CR-E	2019 ^3^	Inhibition of cell wall synthesis by blockage of PBPs (associated BLI protects from inactivation by class A, C and some D β-lactamases)
Eravacycline	Tetracycline	CR-E	2018	Inhibition of protein synthesis at site A of 30S ribosomal subunit
Omadacycline	Tetracycline	MRSA, *Streptococcus pneumoniae* PNS	2018	Inhibition of protein synthesis at site A of 30S ribosomal subunit
Cefiderocol	β-Lactam (Cephalosporin)	CR-E, CR-PA, CR-AB	2019	Siderophore, entering through iron transport systems ^6^, further inhibiting cell wall synthesis by blockage of PBPs
Plazomicin	Aminoglycoside	CR-E	2018	Distortion of 30S ribosomal subunit, leading to production of abnormal proteins that modify cytoplasmic membrane permeability
Sulbactam/Durlobactam ^1b^	β-lactam-β-lactamase inhibitor/Diazabicyclooctane β-lactamase inhibitor	CR-AB	2023	Inhibition of cell wall synthesis by blockage of PBP3 and protection from inactivation by class A, C and D β-lactamases
Pretomanid	Nitroimidazole	Pre-XDR *Mycobacterium tuberculosis*	2019	Inhibition of mycolic acids synthesis and toxic action on the respiratory chain reducing intracellular ATP levels
Contezolid	Oxazolidinone	MRSA	2021 ^3,4^	Inhibition of protein synthesis at 50S ribosomal subunit, by preventing the formation of the 70S initiation complex
Lefamulin	Pleuromutilin	*S. pneumoniae* PNS, *Haemophilus influenzae* AR	2019	Inhibition of protein synthesis at peptidyl transferase center of 50S ribosomal subunit, by preventing elongation

^1a^ New antibiotic combination consisting of the association of a β-lactam antibiotic with a β-lactamase inhibitor; ^1b^ New antibiotic combination consisting of the association of two β-lactamase inhibitors. ^2^ MRSA, methicillin-resistant *Staphylococcus aureus*; CR-E, carbapenem-resistant-*Enterobacterales*; ESBL-E, extended-spectrum-β-lactamase-producing- *Enterobacterales*; PNS, penicillin-non-susceptible; CR-PA, carbapenem-resistant-*Pseudomonas aeruginosa*; CR-AB, carbapenem-resistant-*Acinetobacter baumannii*; pre-XDR, pre-extensively-drug-resistant; AR, ampicillin-resistant. ^3^ FDA-Qualified Infectious Disease Product (QIDP) and Fast Track designation. ^4^ Approved for clinical use in China. ^5^ BLI, β-lactamase inhibitor; PBPs, penicillin-binding proteins. ^6^ New mechanism of entry into bacteria.

**Table 2:** Categories of treatment approaches for multidrug-resistant (MDR) bacteria.

Category	Description
Traditional antibiotic approaches	New antibiotics from previously approved classes of antimicrobials
Non-traditional antibiotic approaches	New antibiotics with new mechanisms of action and/or bacterial targets
Non-antibiotic approaches	Molecules with different modes of action compared to the direct-acting antibiotics (e.g., by inhibiting virulence, boosting the immune system, restoring gut microbiome)

## Data Availability

Not applicable.
